# The relationship between white matter microstructure and self-perceived cognitive decline

**DOI:** 10.1016/j.nicl.2021.102794

**Published:** 2021-08-28

**Authors:** Derek B. Archer, Elizabeth E. Moore, Ujwala Pamidimukkala, Niranjana Shashikumar, Kimberly R. Pechman, Kaj Blennow, Henrik Zetterberg, Bennett A. Landman, Timothy J. Hohman, Angela L. Jefferson, Katherine A. Gifford

**Affiliations:** a Vanderbilt Memory and Alzheimer’s Center, Vanderbilt University Medical Center, Nashville, TN, USA; b Department of Neurology, Vanderbilt University Medical Center, Nashville, TN, USA; c Vanderbilt Genetics Institute, Vanderbilt University School of Medicine, Nashville, TN, USA; d Vanderbilt University Institute of Imaging Science, Vanderbilt University Medical Center, Nashville, TN, USA; e Department of Biomedical Engineering, Vanderbilt University, Nashville, TN, USA; f Department of Electrical and Computer Engineering, Vanderbilt University, Nashville, TN, USA; g Department of Psychiatry and Neurochemistry, Institute of Neuroscience and Physiology, The Sahlgrenska Academy at University of Gothenburg, Mölndal, Sweden; h Clinical Neurochemistry Laboratory, Sahlgrenska University Hospital, Mölndal, Sweden; i Department of Neurodegenerative Disease, University College London Institute of Neurology, Queen Square, London, England; j UK Dementia Research Institute, London, England; k Department of Medicine, Vanderbilt University Medical Center, Nashville, TN, USA

## Abstract

Subjective cognitive decline (SCD) is a perceived cognitive change prior to objective cognitive deficits, and although it is associated with Alzheimer’s disease (AD) pathology, it likely results from multiple underlying pathologies. We investigated the association of white matter microstructure to SCD as a sensitive and early marker of cognitive decline and quantified the contribution of white matter microstructure separate from amyloidosis. Vanderbilt Memory & Aging Project participants with diffusion MRI data and a 45-item measure of SCD were included [n = 236, 137 cognitively unimpaired (CU), 99 with mild cognitive impairment (MCI), 73 ± 7 years, 37% female]. A subset of participants (64 CU, 40 MCI) underwent a fasting lumbar puncture for quantification of cerebrospinal fluid (CSF) amyloid-β(CSF Aβ_42_), total tau (CSF t-tau), and phosphorylated tau (CSF p-tau). Diffusion MRI data was post-processed using the free-water (FW) elimination technique, which allowed quantification of extracellular (FW) and intracellular compartment (fractional anisotropy, mean diffusivity, axial diffusivity, and radial diffusivity) microstructure. Microstructural values were quantified within 11 cognitive-related white matter tracts, including medial temporal lobe, frontal transcallosal, and fronto-parietal tracts using a region of interest approach. General linear modeling related each tract to SCD scores adjusting for age, sex, race/ethnicity, education, Framingham Stroke Risk Profile scores, *APOE* ε4 carrier status, diagnosis, Geriatric Depression Scale scores, hippocampal volume, and total white matter volume. Competitive models were analyzed to determine if white matter microstructural values have a unique role in SCD scores separate from CSF Aβ_42_. FW-corrected radial diffusivity (RD_T_) was related to SCD scores in 8 tracts: cingulum bundle, inferior longitudinal fasciculus, as well as inferior frontal gyrus (IFG) pars opercularis, IFG orbitalis, IFG pars triangularis, tapetum, medial frontal gyrus, and middle frontal gyrus transcallosal tracts. While CSF Aβ_42_ was related to SCD scores in our cohort (R_adj_^2^ = 39.03%; β = −0.231; p = 0.020), competitive models revealed that fornix and IFG pars triangularis transcallosal tract RD_T_ contributed unique variance to SCD scores beyond CSF Aβ_42_ (R_adj_^2^ = 44.35% and R_adj_^2^ = 43.09%, respectively), with several other tract measures demonstrating nominal significance. All tracts which demonstrated nominal significance (in addition to covariates) were input into a backwards stepwise regression analysis. ILF RD_T_, fornix RD_T_, and UF FW were best associated with SCD scores (R_adj_^2^ = 46.69%; p = 6.37 × 10^-12^). Ultimately, we found that medial temporal lobe and frontal transcallosal tract microstructure is an important driver of SCD scores independent of early amyloid deposition. Our results highlight the potential importance of abnormal white matter diffusivity as an early contributor to cognitive decline. These results also highlight the value of incorporating multiple biomarkers to help disentangle the mechanistic heterogeneity of SCD as an early stage of cognitive decline.

## Highlights

• RD_T_ within several white matter tracts is associated with SCD.
• RD_T_ contributes unique variance to SCD beyond that of CSF Aβ_42_.
• Our findings suggest that RD_T_ is a sensitive marker of SCD.

## 1 Introduction

Subjective cognitive decline (SCD) is an individual’s self-report of decline or change to cognition (Jessen et al., 2014) and is thought to occur prior to the onset of objective cognitive deficits (Sperling et al., 2011). SCD is a measure which can be reliably measured and is associated with future cognitive changes (Gifford et al., 2014) and conversion to dementia (Gifford et al., 2014), suggesting it may be one of the earliest detectable changes of unhealthy brain aging. SCD is associated with Alzheimer’s disease (AD) neuroimaging biomarkers (Lin et al., 2019) (e.g., temporal lobe cortical thinning, (Verfaillie et al., 2018, Meiberth et al., 2015) smaller hippocampal volume (van der Flier et al., 2004, Cherbuin et al., 2015)) and greater SCD is also associated with increased amyloid- β (Aβ) load (Verfaillie et al., 2019, Perrotin et al., 2017). While SCD may be relevant in early AD, SCD is a heterogenous construct, likely representing the culmination of many etiologies (Perrotin et al., 2017).

Abnormal white matter microstructure, particularly in the medial temporal lobe projections and frontal lobe tracts, is associated with SCD, with prior literature emphasizing contributions of hippocampal projections, forceps minor, forceps major, the inferior longitudinal fasciculus, and the cingulum bundle (Luo et al., 2019, Ohlhauser et al., 2019). Individuals with SCD have white matter microstructural damage similar to patients with mild cognitive impairment (MCI) (Luo et al., 2019) and diffusion MRI metrics may be more sensitive to SCD than volumetric analyses (Ryu et al., 2017). White matter abnormalities may precede atrophic processes, (Metzler-Baddeley et al., 2019, Bartzokis, 2011) represent a more sensitive marker of early clinical symptoms, (Selnes et al., 2013) and are associated with multiple injury pathways in aging (e.g., cerebrovascular disease) (de Groot et al., 2015). Thus, white matter integrity may be a suitable biomarker for SCD.

However, conventional diffusion MRI techniques have limitations, (Jones et al., 2013) such as partial volume effects, (Pasternak et al., 2009, Alexander et al., 2001) making it difficult to distinguish between intracellular and extracellular compartments. New post-processing techniques, (Pasternak et al., 2009, Pasternak et al., 2012) such as free-water (FW) elimination, separate the diffusion MRI image into intracellular and extracellular compartments. Specifically, the FW-elimination technique is a validated technique which uses a bi-tensor mathematical framework to estimate the proportion of each voxel which has unrestricted diffusion. This proportion of the voxel is then removed from the voxel to provide more accurate assessments of the underlying tissue. Increases in the extracellular compartment are thought to be related to inflammation, atrophy, and edema, while alterations of the intracellular component are thought to be associated with tissue degeneration (Pasternak et al., 2009, Gullett et al., 2020, Pasternak et al., 2016). Using this technique, reductions in white matter microstructure have been associated with higher levels of AD pathology in preclinical AD (Hoy et al., 2017) and were more closely associated with AD pathology than conventional diffusion MRI metrics (Walsh et al., 2020). Finally, our group recently demonstrated that FW-corrected measures interacted with hippocampal atrophy to predict more rapid rates of cognitive decline (Archer et al., 2020). While FW-corrected metrics have been leveraged to evaluate several aspect of objective cognitive decline, they have not yet been used to assess SCD.

The purpose of this study is to use FW post-processing techniques to investigate the associations between white matter microstructure and SCD scores among older adults without clinical dementia. We therefore evaluated white matter tracts which have demonstrated an association with cognitive impairment, which include those projecting from the prefrontal cortex and medial temporal lobe. The involvement of these tracts may arise from a variety of factors, including normal aging, AD, and other pathologies (e.g., vascular disease) (Luo et al., 2019, Ohlhauser et al., 2019, Archer et al., 2020, Archer et al., 2019). These tracts included five medial temporal lobe projections (cingulum bundle, tapetum, fornix, uncinate fasciculus, inferior longitudinal fasciculus), one fronto-parietal association tract (superior longitudinal fasciculus), and five frontal homologous transcallosal tracts (medial frontal gyrus, middle frontal gyrus, and inferior frontal gyrus pars opercularis, orbitalis, and triangularis). We then quantified FW-corrected metrics within these tracts, including fractional anisotropy (FA_T_), mean diffusivity (MD_T_), axial diffusivity (AD_T_), and radial diffusivity (RD_T_) using a region-of-interest (ROI) based approach. These microstructural metrics were associated with SCD scores derived from a 45-item questionnaire of self-reported cognitive decline considered on a continuous spectrum (Gifford et al., 2015). We hypothesized that abnormalities in white matter tract diffusivity would be associated with higher SCD scores. We also examined whether these metrics were independently associated with SCD scores beyond CSF Aβ_42_. Finally, multivariate regression was conducted to determine the subset of variables which was most associated with SCD scores.

## 2 Materials & methods

### 2.1 Study cohort

Data were drawn from the baseline cohort of the Vanderbilt Memory & Aging Project (VMAP), which is a longitudinal observational study of 335 individuals without dementia launched in 2012 and described elsewhere (Jefferson et al., 2016). Briefly, inclusion criteria for VMAP required participants to be 60 years of age and older, speak English, have adequate auditory/visual acuity, and have a dependable study partner. Participants underwent a neuropsychological assessment for cognitive diagnosis by consensus. Specifically, participants were considered cognitively unimpaired (CU) if they had a Clinical Dementia Rating (CDR) scale score equal to 0, no deficits in activities of daily living attributable to cognitive impairment, (Pfeffer et al., 1982) and had neuropsychological impairment scores within 1.5 standard deviations of the age-adjusted normative mean. In contrast, individuals were considered to have mild cognitive impairment (MCI) if they had a (1) a CDR score ≥ 0.5, (2) relatively spared activities of daily living, (3) neuropsychological performance more than 1.5 standard deviations from the group neuropsychological performance mean in at least one cognitive domain, (4) concern of a cognitive change by the individual, an information, or a clinician, and (5) absence of dementia (Morris, 1993, Albert et al., 2011). Participants were excluded from the VMAP cohort for a variety of well-defined reasons, including contraindication for 3 T MRI, a history of other neurological disorders (e.g., dementia, stroke, major psychiatric illness), heart failure, head injury with 5 min or more of unconsciousness, and systemic or terminal illness. In the present study, participants were also excluded if they were missing covariates, SCD assessment, or diffusion MRI data, resulting in a sample of 236 individuals in our primary analysis (137 CU, 99 MCI). Further, we conducted an analysis leveraging cerebrospinal fluid in our cohort. In this analysis, there were 104 individuals (64 CU, 40 MCI). The protocol was approved by the Vanderbilt University Medical Center Institutional Review Board, and written informed consent was obtained prior to data collection for all participants.

### 2.2 Subjective cognitive decline (SCD) assessment

Participants completed four SCD questionnaires: the Everyday Cognition Questionnaire (Farias et al., 2008), the Memory Functioning Questionnaire (Gilewski et al., 1990), the Cognitive Changes Questionnaire (Farias et al., 2008, Gilewski et al., 1990, Crook, 1983, Reid and MacLullich, 2006), and the Cognitive Difficulties Scale (Crook, 1983). Items from these questionnaires were reduced into a 45-item questionnaire (Vanderbilt SCD Questionnaire) using factor analytic models (Gifford et al., 2015). Total score on this measure ranges from 5 to 174, with higher scores representing more SCD. This study considered the SCD score as a continuous marker of subjective cognitive experience, rather than a dichotomized diagnostic status. We considered SCD status across the cognitive spectrum, including CU and MCI individuals (Gifford et al., 2015). Moreover, this score can be partitioned into three subscores: memory, executive function, and language.

### 2.3 Diffusion MRI acquisition and preprocessing

Diffusion images (resolution: 2 mm isotropic, *b*-values: 0, 1000 s/mm^2^) were collected along 32 diffusion gradient vectors and 1 B_0_ weighted image. FSL 5.0.9 (fsl.fmrib.ox.ax.uk) was used for all diffusion MRI preprocessing (Jenkinson et al., 2012). Quality assessment of all diffusion MRI scans was performed manually. Data were first corrected for head motion and eddy currents and the brain was then extracted from the skull (Smith, 2002, Andersson and Sotiropoulos, 2016). This corrected image was then inputted into custom written MATLAB (R2019a; The MathWorks, Natick, MA) to calculate free-water corrected fractional anisotropy (FA_T_), mean diffusivity (MD_T_), axial diffusivity (AD_T_), radial diffusivity (RD_T_), and FW maps, as previously described (Archer et al., 2020, Archer et al., 2019). The diffusion images were then standardized to the Montreal Neurological Institute (MNI) space using the FA_T_ maps. Specifically, the FA_T_ map was registered to an in-house template (1 mm isotropic) by a non-linear warp using the Advanced Normalization Tools (ANTs) package (Avants et al., 2008). Normalization accuracy was assessed manually. This non-linear warp was then applied to the MD_T_, AD_T_, RD_T_, and FW maps.

**Fig. 1:**
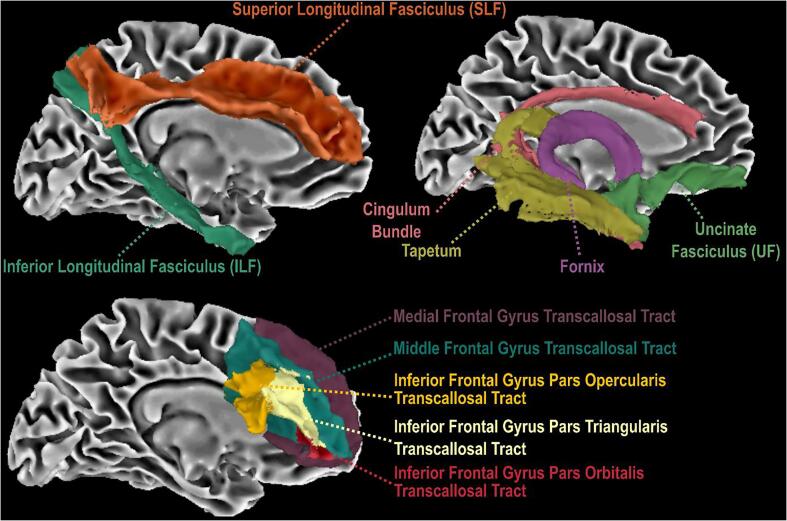
**Tractography Templates Used in Study.** This study used several previously developed tractography templates to evaluate tract microstructure. All tractography templates are freely available at: https://github.com/VUMC-VMAC/Tractography_Templates. (Archer et al., 2020, Archer et al., 2019, Brown et al., 2017).

A set of 11 white matter tractography templates in MNI space were collated from several well-established templates, (Archer et al., 2020, Brown et al., 2017, DeSimone et al., 2019) and included medial temporal lobe projections, frontal transcallosal tracts, and fronto-parietal association tracts (Fig. 1). Frontal transcallosal tracts included homologous connections of the inferior frontal gyrus (IFG) pars opercularis, orbitalis, and triangularis as well as inferior temporal gyrus (i.e., tapetum), medial frontal gyrus, and middle frontal gyrus. The fronto-parietal association tract included the superior longitudinal fasciculus (SLF). Medial temporal projections included the cingulum bundle, fornix, inferior longitudinal fasciculus (ILF), and uncinate fasciculus (UF). Using a region-of-interest (ROI) based approach, FA_T_, MD_T_, AD_T_, RD_T_, and FW metrics were the calculated within all 11 tracts for all participants, resulting in a total of 55 values for each participant.

### 2.4 Cerebrospinal fluid acquisition

A subset of participants completed a fasting lumbar puncture (n = 104). CSF was collected with polypropylene syringes using a Sprotte 25-gauge spinal needle in an intervertebral lumbar space. Samples were immediately mixed and centrifuged, and supernatants were aliquoted in 0.5 mL polypropylene tubes and stored at −80 °C. Samples were analyzed in batch using commercially available enzyme-linked immunosorbent assays (Fujirebio, Ghent, Belgium) to determine the levels of Aβ_42_ (INNOTEST β-AMYLOID_(1–42)_), p-tau (INNOTEST PHOSPHO-TAU_(181P)_), and t-tau (INNOTEST hTAU) (Osborn et al., 2018). Board-certified laboratory technicians processed data blinded to clinical information, as previously described (Palmqvist et al., 2014).

### 2.5 Analytical plan

Covariates included age, sex, race/ethnicity, education, Framingham Stroke Risk Profile (FSRP) Score, (Wolf et al., 1991, D'Agostino et al., 1994) apolipoprotein E (*APOE-*ε4) carrier status, diagnosis, and Geriatric Depression Score (GDS) (Yesavage, 1988). Given that we were interested in the association of white matter and SCD scores beyond that of AD contributors, we also adjusted our models for hippocampal volume using established procedures (Archer et al., 2020). Finally, since the temporal ordering of gray and white matter volume loss is currently unclear (Sachdev et al., 2013), we included a white matter volume covariate. *APOE-* ε*4* genotype status (ε2, ε3, ε4) was determined using single-nucleotide polymorphisms rs7412 and rs429358, which were genotyped using a TaqMan from DNA extracted from frozen whole blood (Jefferson et al., 2016). *APOE-*ε4 carrier status was defined as positive (ε2/ε4, ε3/ε4, ε4/ε4) or negative (ε2/ε2, ε3/ε3, ε2/ε3). For primary models, the effect of each tract (IFG pars opercularis, IFG pars orbitalis, IFG pars triangularis, tapetum, medial frontal gyrus, middle frontal gyrus, cingulum bundle, fornix, ILF, SLF, and UF) for all five measures (FA_T_, MD_T_, AD_T_, RD_T_, and FW) on total SCD score was estimated using a general linear model adjusting for all covariates. For each analysis, only one tract per model was considered, and models were standardized to allow easy comparison of beta-coefficients between microstructural measures. Next, the association between CSF Aβ_42_ and SCD scores was estimated using a general linear model adjusting for all covariates in a subset of our cohort which had both diffusion MRI and CSF Aβ_42_ data (n = 104; 64 CU, 40 MCI). Secondary analyses were conducted to determine if diffusion MRI and CSF measures interacted on SCD scores.

In a separate analysis in the CSF cohort, we then iteratively added each white matter tract metric to determine if unique variance was provided above and beyond covariates and CSF Aβ_42_ measures (i.e., a competitive model analysis). Following this analysis, we collated variables which significantly added unique variance above and beyond CSF Aβ_42_ and conducted a multivariate regression analysis. Specifically, these input variables (in addition to covariates) were inputted into a backwards stepwise regression analysis to determine the set of variables which maximized the R_adj_^2^. In the case where RD_T_ and MD_T_ were significant for a particular tract, the MD_T_ measure was excluded to avoid collinearity. Significance was set a priori as α = 0.05 and correction for multiple comparisons were made using the false discovery rate (FDR) method across all tracts for each measure. All statistical analyses were performed in R version 3.5.2. (http://www.r-project.org/).

## 3 Results

### 3.1 Participant characteristics

Demographic and clinical information for each group (CU, MCI) are summarized in Table 1. As expected, the MCI group had lower MoCA scores and higher SCD scores compared to the cognitively unimpaired group. The MCI group had higher SCD scores, and these findings were consistent across all subscores of SCD (i.e., executive function, language, memory). CSF biomarker data for the CSF cohort (n = 104) is also shown in Table 1. The MCI group had significantly lower CSF Aβ_42_ and higher CSF t-tau. There were no between group differences in CSF p-tau. Demographic and clinical information for the CSF cohort can be found in Supplemental Table 1.

**Table 1:** Participant Demographic and Clinical Characteristics.

Measure	Cognitive Status			Test Statistic	*p*-value
	All	Cognitively Unimpaired (CU)	MCI		
*Demographics*					
Sample size	236	137	99	–	–
Age (years)	73 (7)	73 (7)	73 (8)	t = 0.794	0.480
Sex (% male)	63	64	62	χ^2^ = 0.075	0.784
Education (years)	16 (3)	16 (3)	15 (3)	t = 3.680	**<0.001**
Race (% Non-Hispanic white)	86	85	88	χ^2^ = 0.127	0.722

*Clinical Characteristics*					
MoCA	25 (3)	27 (2)	23 (3)	H = 72.786	**<0.001**
*APOE* ε4 (% positive)	35	41	30	χ^2^ = 2.857	0.096
FSRP Score^a^	12 (4)	12 (4)	13 (4)	t = 1.248	0.213
Systolic Blood Pressure (mmHg)	142 (18)	141 (18)	145 (19)	t = 1.522	0.129
Prevalent CVD (%)	5	5	3	χ^2^ = 0.486	0.486
GDS Score^b^	2 (3)	2 (2)	3 (3)	H = 17.476	**<0.001**
Hippocampal Volume (mm^3^)	7228 (7 6 7)	7459 (6 7 6)	6907 (7 7 3)	t = 5.817	**<0.001**

*CSF Biomarkers^c^*					
Aβ_42_ (pg/mL)	706 (241)	760 (231)	620 (234)	t = 2.996	**0.003**
T-tau (pg/mL)	422 (212)	383 (180)	486 (244)	H = 4.416	**0.036**
P-tau (pg/mL)	61 (26)	57 (22)	67 (29)	H = 2.573	0.109

*Subjective Cognitive Decline Scores*					
Total Score	62 (23)	52 (17)	75 (22)	H = 57.613	**<0.001**
Executive Function	11 (5)	9 (4)	14 (6)	H = 40.988	**<0.001**
Language	14 (6)	12 (4)	17 (7)	H = 44.076	**<0.001**
Memory	37 (13)	32 (10)	44 (12)	H = 8.686	**<0.001**

Values denoted as mean (standard deviation) or frequency. Abbreviations: MoCA, Montreal Cognitive Assessment; *APOE* ε4, apolipoprotein E ε4; CVD, cardiovascular disease; FSRP, Framingham Stroke Risk Profile; GDS, Geriatric Depression Scale; MCI, mild cognitive impairment. p-values (p < 0.05 bolded) were generated using a one-way analysis of variance for continuous variables and a chi-square test for categorical variables. ^a^A modified FSRP score was included in statistical models excluding points assigned to age. ^b^GDS score minus points from items 14, 26, 29, and 30. The adjusted range of this score is 0–26. ^c^A subset of 104 participants (64 CU, 40 MCI) had CSF biomarker data. Bolded values indicate p < 0.05.

### 3.2 White matter tract microstructure association with subjective cognitive decline scores

**Fig. 2:**
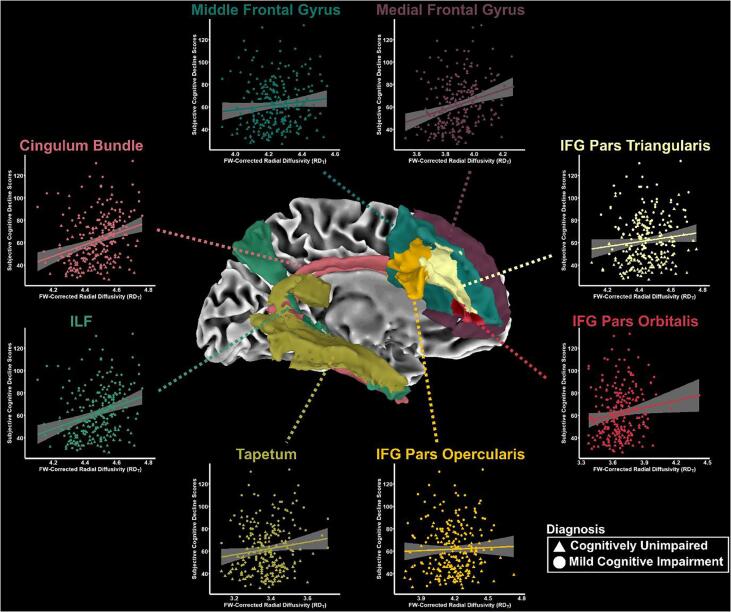
**RD_T_ Associations with Subjective Cognitive Decline (SCD) scores.** (**A**) RD_T_ within the cingulum bundle, medial frontal gyrus, middle frontal gyrus, inferior longitudinal fasciculus (ILF), IFG pars triangularis, IFG pars orbitalis, IFG pars opercularis, and tapetum were significant predictors of subjective cognitive decline. (**B**) Solid lines reflect fitted regressions with tract RD_T_ and SCD. Shading reflects 95% CI.

The association of FW and FW-corrected white matter tract microstructure (FW, FA_T_, MD_T_, AD_T_, RD_T_) with SCD scores are presented in Table 2 and illustrated in Fig. 2. For FW, there were no significant associations which survived multiple correction. For FA_T_, significant associations were found for the cingulum bundle, ILF, IFG pars orbitalis, IFG pars triangularis, medial frontal gyrus, middle frontal gyrus, SLF, and UF. For MD_T_, there were no significant associations which survived multiple correction. Higher RD_T_ in the cingulum bundle, ILF, IFG pars opercularis, IFG pars orbitalis, IFG pars triangularis, tapetum, medial frontal gyrus, and middle frontal gyrus was significantly associated with higher SCD scores. Secondary analyses were conducted to determine the association of tract measures with each SCD subscore. These findings can be found in Supplemental Tables 2-4 and demonstrate that memory and executive function subscores are largely similar to total SCD score associations; in contrast, associations with language were less significant. Further, we conducted an analysis to determine if diffusion MRI and CSF biomarkers interacted on SCD scores. No significant associations were found.

**Table 2:** White Matter Tract Microstructure Associations with Subjective Cognitive Decline.

	Cingulum Bundle	Fornix	ILF	IFG Pars Opercularis	IFG Pars Orbitalis	IFG Pars Triangularis	Tapetum	Medial Frontal Gyrus	Middle Frontal Gyrus	SLF	UF
*Free-water (FW)*											
**β**	0.043	0.009	0.058	0.069	0.105	0.062	0.084	0.127	0.091	0.066	0.140
**β_SE_**	0.074	0.071	0.071	0.068	0.067	0.067	0.065	0.062	0.068	0.065	0.067
**p-value**	0.565	0.901	0.410	0.314	0.116	0.355	0.202	**0.042**	0.184	0.306	**0.037**
**f^2^**	0.001	0.000	0.003	0.005	0.011	0.004	0.007	0.019	0.008	0.005	0.020

*FW-corrected fractional anisotropy (FA_T_)*											
**β**	−0.168	0.000	−0.186	−0.146	−0.136	−0.153	−0.115	−0.153	−0.150	−0.121	−0.139
**β_SE_**	0.058	0.062	0.058	0.053	0.057	0.054	0.057	0.059	0.055	0.055	0.057
**p-value**	0.004*	1.000	0.001*	**0.007**	0.018*	0.005*	**0.043**	0.011*	0.007*	0.028*	0.016*
**f^2^**	0.037	0.000	0.046	0.033	0.025	0.035	0.018	0.030	0.033	0.022	0.026

*FW-corrected mean diffusivity (MD_T_)*											
**β**	0.154	0.067	0.114	0.112	0.035	0.118	0.082	0.143	0.106	0.038	0.034
**β_SE_**	0.055	0.067	0.053	0.056	0.054	0.056	0.055	0.052	0.056	0.055	0.057
**p-value**	**0.005**	0.314	**0.033**	**0.047**	0.520	**0.038**	0.134	**0.007**	0.060	0.492	0.550
**f^2^**	0.035	0.005	0.020	0.018	0.002	0.020	0.010	0.033	0.016	0.002	0.002

*FW-corrected axial diffusivity (AD_T_)*											
**β**	−0.080	0.010	−0.114	−0.075	−0.119	−0.106	−0.038	−0.096	−0.100	−0.100	−0.115
**β_SE_**	0.065	0.065	0.061	0.055	0.054	0.056	0.055	0.057	0.055	0.058	0.061
**p-value**	0.220	0.878	0.061	0.171	**0.029**	0.058	0.492	0.093	0.072	0.087	0.061
**f^2^**	0.007	0.000	0.016	0.008	0.021	0.016	0.002	0.013	0.015	0.013	0.016

*FW-corrected radial diffusivity (RD_T_)*											
**β**	0.181	0.075	0.173	0.148	0.136	0.161	0.121	0.175	0.154	0.087	0.110
**β_SE_**	0.052	0.060	0.053	0.054	0.053	0.052	0.053	0.053	0.053	0.053	0.054
**p-value**	0.001*	0.211	0.001*	0.006*	0.010*	0.002*	0.024*	0.001*	0.004*	0.100	**0.042**
**f^2^**	0.054	0.007	0.047	0.034	0.030	0.042	0.023	0.048	0.038	0.012	0.019

FW-corrected metrics were associated with SCD in 236 participants (137 CU, 99 MCI). Abbreviations: IFG, inferior frontal gyrus; ILF, inferior longitudinal fasciculus; SLF, superior longitudinal fasciculus; UF, uncinate fasciculus; β, beta-coefficient for variable of interest; β_SE_, standard error of the beta-coefficient. Bolded values indicate p < 0.05, *p_FDR_ < 0.05.

### 3.3 CSF Aβ_42_ and white matter tract microstructure as competitive predictors of subjective cognitive decline scores

The main effect analyses were followed up with analyses in a subset of our cohort which had both diffusion MRI and CSF Aβ_42_ data (n = 104). We recapitulate previous findings (Stomrud et al., 2007); (Wolfsgruber et al., 2015) by demonstrating that CSF Aβ_42_ is associated with SCD scores (β = −0.231, p = 0.020), whereas t-tau (β = 0.138, p = 0.122) and p-tau (β = 0.129, p = 0.141) are not associated with SCD scores. Therefore, we further assessed white matter associations above and beyond the established associations with CSF Aβ_42_ (Table 3). The base model (i.e., CSF Aβ_42_ and all covariates) explained 39.03% of the variance to SCD scores (p = 1.78 × 10^-8^). White matter tract microstructural measures were then iteratively added to this model. We found that the fornix MD_T_, fornix RD_T_, and IFG pars triangularis RD_T_ contributed unique variance beyond the base model. Specifically, the inclusion of fornix MD_T_, fornix RD_T,_ and IFG pars triangularis RD_T_ to the base model results increased the R_adj_^2^ by 5.44%, 5.32%, and 4.06%, respectively.

**Table 3:** Competitive Models of White Matter Microstructure and Amyloidosis with Subjective Cognitive Decline.

Model	Δ R_adj_^2^				
	FW	FA_T_	MD_T_	AD_T_	RD_T_
Cingulum Bundle	−0.481	1.581	0.802	−0.195	1.854
Fornix	0.613	−0.003	5.438*	0.977	5.318*
ILF	−0.226	1.440	0.728	0.303	**2.389**
IFG Pars Opercularis	−0.327	0.497	**2.523**	−0.571	**1.965**
IFG Pars Orbitalis	−0.328	1.640	−0.455	1.421	**2.310**
IFG Pars Triangularis	−0.558	1.541	**3.523**	0.068	4.058*
Tapetum	−0.015	0.021	1.806	−0.536	**2.229**
Medial Frontal Gyrus	−0.160	0.188	**2.321**	−0.091	**2.269**
Middle Frontal Gyrus	−0.222	1.695	1.546	0.616	**2.931**
Superior Longitudinal Fasciculus	−0.592	0.336	−0.645	0.065	−0.208
Uncinate Fasciculus	**2.323**	0.379	−0.413	0.676	−0.346

The baseline model (CSF Aβ_42_ and all covariates) explained 39.03% of the variance to SCD in 104 participants (64 CU, 40 MCI). White matter tract microstructural values were then iteratively added to this base model and the R_adj_^2^ values were compared. The table shows the ΔR_adj_^2^ for each model. Abbreviations: AD_T_, free-water corrected axial diffusivity; FA_T_, free-water corrected fractional anisotropy; FW, free-water; IFG, inferior frontal gyrus; ILF, inferior longitudinal fasciculus; MD_T_, free-water corrected mean diffusivity; RD_T_, free-water corrected radial diffusivity. Bolded values indicate p-uncorrected < 0.05. *p_FDR_ < 0.05.

### 3.4 Multivariate regression to maximize the association with subjective cognitive decline scores

**Fig. 3:**
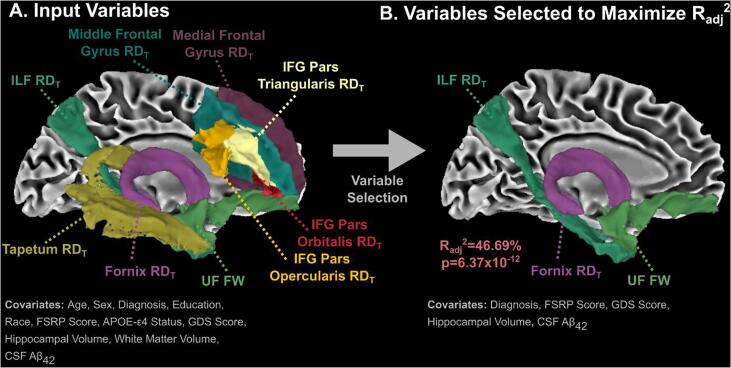
**Multivariate Association with Subjective Cognitive Decline (SCD).** (**A**) Tract measures which demonstrated significance in the competitive model (in addition to covariates) were inputted into backwards stepwise multiple regression analysis to maximize the R_adj_^2^. (**B**) The set of variables which maximized the R_adj_^2^ included inferior longitudinal fasciculus (ILF) RD_T_, fornix RD_T_, uncinate fasciculus (UF) FW, diagnosis, Framingham Stroke Risk Profile (FSRP) score, Geriatric Depression Score (GDS), hippocampal volume, and CSF Aβ_42_.

All variables which were nominally significant (p < 0.05) in the competitive model analysis, in addition to covariates, were then selected as input variables in a backwards stepwise multiple regression analysis to maximize the R_adj_^2^ value (Fig. 3**A**). The set of variables which resulted in the best model (R_adj_^2^ = 46.69%; p = 6.37 × 10^-12^) included ILF RD_T_, fornix RD_T_, UF FW, diagnosis, FSRP score, GDS score, hippocampal volume, and CSF Aβ_42_ (Fig. 3**B**). This model increased the R_adj_^2^ by 7.66% compared to the base model (i.e., covariates + CSF Aβ_42_ only).

## 4 Discussion

The current study leveraged high-resolution white matter tractography templates in conjunction with an advanced diffusion MRI post-processing technique (i.e., FW elimination) to investigate if white matter tract microstructure is associated with SCD. Moreover, we determined if FW-corrected diffusion MRI metrics accounted for significant variance in association with SCD scores beyond that of amyloidosis (CSF Aβ_42_). First, we found that RD_T_ showed the most widespread associations with SCD scores, and many of these RD_T_ associations explained variance in SCD scores above and beyond CSF Aβ_42_. Second, we conducted a multivariate backwards stepwise regression analysis to find the subset of variables which was best associated with SCD scores, and found that these variables included ILF RD_T_, fornix RD_T_, and UF FW (in addition to several well-established covariates). These findings extend prior work reporting associations between multiple diffusion MRI metrics and SCD (Lin et al., 2019, Luo et al., 2019, Ohlhauser et al., 2019, Selnes et al., 2013, Li et al., 2016, Brueggen et al., 2019). Our findings suggest that white matter microstructural values, particularly RD_T_, could have important contributions to SCD that are independent of early AD pathology.

White matter tract integrity is critical for efficient signal propagation between neurons and damage is associated with cognitive disruption; (Ryu et al., 2017, Selnes et al., 2013, DeSimone et al., 2019, Shu et al., 2018) thus, it is not surprising white matter tract abnormalities have been related to SCD (Lin et al., 2019, Luo et al., 2019, Ohlhauser et al., 2019, Selnes et al., 2013, Li et al., 2016, Brueggen et al., 2019). However, this study was among the first to leverage FW correction to evaluate diffusivity metrics, thus providing more sensitive quantification of tissue abnormalities. Using this method, we demonstrated that RD_T_ may be a stronger correlate of SCD scores than other intracellular diffusion MRI metrics (FA_T_, AD_T_, MD_T_). Although RD_T_ has been implicated in a variety of pathophysiological mechanisms (e.g., demyelination, axonal degeneration, edema) (Winklewski et al., 2018, Song et al., 2003, Song et al., 2005, Kronlage et al., 2017), the prevailing hypothesis is that RD_T_ is strongly associated with demyelination. For example, a histological mouse study found that higher radial diffusivity in the corpus callosum was associated with demyelination but not associated with axonal degeneration (Song et al., 2005). While the underlying pathology remains somewhat unclear, our findings demonstrate a clear association between RD_T_ and SCD scores in older adults; thus, it’s possible that demyelination leads to reduced conduction velocity between critical gray matter regions, ultimately leading to cognitive complaint (Plemel et al., 2017). Future studies which pair *in-vivo* neuroimaging with *post-mortem* histopathological analysis will be pivotal to elucidate the exact pathophysiological mechanisms driving the white matter association with SCD.

The role of white matter tract microstructure in SCD has been investigated in prior studies. The cingulum bundle, forceps major, forceps minor, and ILF are tracts and regions consistently associated with SCD (Luo et al., 2019, Ohlhauser et al., 2019). The current work extends these findings by using FW post-processing techniques and spatially precise templates of cognitive related white matter tracts to implicate several white matter tracts with SCD scores. In our primary analysis, we found widespread FA_T_ and RD_T_ associations with SCD scores. Following these analyses, we conducted a competitive model analysis in our CSF cohort to find that several tracts contributed unique variance beyond that of CSF Aβ_42_. While many tracts demonstrated nominal significance, MD_T_ within the fornix and RD_T_ within the fornix and IFG pars triangularis demonstrated significance after correction for multiple comparisons. In our final analysis, we input all variables which demonstrated nominal significance (in addition to covariates) into a multivariate regression analysis to optimize the association with SCD scores, and found that RD_T_ within the ILF and fornix, in addition to FW within the UF, were tract measures involved in this model. Consistent with prior work, these analyses implicate several frontal and medial temporal lobe projections with SCD (Ohlhauser et al., 2019). Importantly, these tracts are traditionally associated with executive function, (Stuss and Alexander, 2000) information processing speed, (Kochunov et al., 2010) and memory, (Fujie et al., 2008) so damage in these tracts may contribute to SCD through these cognitive domains. Notably, a secondary analysis (Supplemental Tables 2-4) of SCD subscores (i.e., memory, executive function, language) found that these white matter tract measures were more strongly associated with memory and executive function. Our findings also align with our previous work linking medial temporal lobe tracts to executive function and memory domains of objective cognitive function (Archer et al., 2020). However, further research is needed to better understand the regional and mechanistic specificity of these findings as it is unclear whether SCD is specific to certain tracts and types of white matter damage or rather general white matter injury.

Our findings also suggest that the contribution of white matter microstructure to SCD scores is statistically independent from the contribution of Aβ. This finding is particularly notable given that Aβ has been widely associated with SCD (Lin et al., 2019, Selnes et al., 2013, Schoonenboom et al., 2012, Fortea et al., 2011). This finding is consistent with prior work showing that diffusivity metrics are more sensitively associated with cognitive decline and medial temporal lobe atrophy in preclinical AD than CSF Aβ (Selnes et al., 2013). While the pathological mechanism driving the white matter microstructural changes remains unclear, these results provide further evidence that multiple etiologies contribute to SCD and suggest that FW imaging may be a useful biomarker to more sensitively identify individuals with SCD. Notably, the tracts which provided the most unique variance to SCD scores predominantly project to the frontal lobe, which is highly implicated in episodic memory retrieval and an important contributor to SCD (Barredo et al., 2016, Senova et al., 2020). It is important to note that RD_T_ in some tracts (e.g., cingulum bundle) did not contribute unique variance to SCD scores beyond Aβ, suggesting that alterations in particular tracts could more accurately delineate the etiologies underlying SCD. Future work should examine the specific mechanism underlying these changes and if other forms of white matter injury mediate associations between Aβ and SCD. Further, models which incorporate both Aβ and measures of white matter injury should be considered.

In contrast to our widespread findings with RD_T_, there were only subtle associations with AD_T_, suggesting that axonal degeneration may not be a large contributor to SCD (Song et al., 2003). Further, while there were not strong associations with FW, we did find a nominal association of UF FW in our primary analysis as well as involvement in our multivariate regression analysis. FW is a measure which has been consistently used to evaluate extracellular changes in a variety of neurodegenerative diseases and reflects the unrestricted FW within white matter (Pasternak et al., 2009, Pasternak et al., 2012, Pasternak et al., 2016). It has been suggested to be driven by a variety of pathologies (e.g., neuroinflammation) and is consistently shown to be one of the earliest structural brain changes on the AD continuum (Hoy et al., 2017, Akiyama et al., 2000). Together, our results strongly suggest that RD_T_ may be useful as an imaging marker, in addition to Aβ or other molecular biomarkers, to better understand the multiple etiologies underlying SCD (e.g., normal aging, vascular disease, AD pathology). Additionally, our results suggest a potential role of UF FW with SCD. Further research leveraging large-scale neuroimaging data could further clarify the role of these metrics with SCD.

The present study has several strengths. First, we leveraged high-resolution white matter tractography templates of the homologous frontal transcallosal tracts, medial temporal lobe projections, and a parieto-frontal tract. The intention of using these tractography templates is that it makes results more consistent and generalizable across studies. Importantly, these tractography templates are freely available to the public. Second, we paired these tractography templates with the FW post-processing technique, which allowed for the separation of extracellular and intracellular components of the diffusion image. Third, these neuroimaging analysis novelties were leveraged to study SCD scores in the Vanderbilt Memory and Aging Project (VMAP) cohort, which is a well-established longitudinal cohort of aging (Jefferson et al., 2016) with comprehensive covariate collection. Despite these strengths, the VMAP cohort is mostly male, non-Hispanic white and well-educated; therefore, generalizability outside of this population may be limited. Another potential limitation of this study is that it leveraged an ROI-based approach of white matter tracts. It’s possible that more comprehensive methods, such as tract-based spatial statistics (TBSS) (Smith et al., 2006), may provide different results. Additionally, only a subset of our cohort (n = 104) had both diffusion MRI and CSF data, thus our analyses using both measures may be underpowered. Finally, the inclusion of multiple comparisons in our study may increase the possibility of false positives, though this was mitigated with false discovery correction. Replication of results in a larger, more diverse cohort is needed to further elucidate the association between white matter tract microstructure and SCD.

In conclusion, this study provided novel evidence that FW-corrected metrics within frontal transcallosal tracts, medial temporal lobe projections, and fronto-parietal tracts are associated with SCD scores, a purported marker of early neurodegenerative changes. Our study found particularly robust associations of FW-corrected radial diffusivity measures on SCD scores, which had unique contributions beyond that of amyloidosis. These findings indicate that early demyelination of white matter is a statistically independent contributor to SCD in older adults. A more robust consideration of both AD pathology and white matter microstructure may help disentangle the mechanistic heterogeneity of SCD as an early stage of cognitive decline.

## 5 Funding acknowledgements

IIRG-08–88733, R01-AG034962, R01-AG059716, K01-AG049164, R01-AG056534, K24-AG046373, F30-AG064847, T32-GM007347, P20-AG068082, K23 AG045966, R01AG062826. KB is supported by the Swedish Research Council (#2017–00915), the Alzheimer Drug Discovery Foundation (ADDF), USA (#RDAPB-201809–2016615), the Swedish Alzheimer Foundation (#AF-742881), Hjärnfonden, Sweden (#FO2017-0243), the Swedish state under the agreement between the Swedish government and the County Councils, the ALF-agreement (#ALFGBG-715986), the European Union Joint Program for Neurodegenerative Disorders (JPND2019-466–236), and the National Institute of Health (NIH), USA, (grant #1R01AG068398-01). HZ is a Wallenberg Scholar supported by grants from the Swedish Research Council (#2018–02532), the European Research Council (#681712), Swedish State Support for Clinical Research (#ALFGBG-720931), the Alzheimer Drug Discovery Foundation (ADDF), USA (#201809–2016862), the AD Strategic Fund and the Alzheimer's Association (#ADSF-21–831376-C, #ADSF-21–831381-C and #ADSF-21–831377-C), the Olav Thon Foundation, the Erling-Persson Family Foundation, Stiftelsen för Gamla Tjänarinnor, Hjärnfonden, Sweden (#FO2019-0228), the European Union’s Horizon 2020 research and innovation programme under the Marie Skłodowska-Curie grant agreement No 860,197 (MIRIADE), and the UK Dementia Research Institute at UCL.

## 6 Disclosure Statement

KB has served as a consultant, at advisory boards, or at data monitoring committees for Abcam, Axon, Biogen, JOMDD/Shimadzu. Julius Clinical, Lilly, MagQu, Novartis, Roche Diagnostics, and Siemens Healthineers, and is a co-founder of Brain Biomarker Solutions in Gothenburg AB (BBS), which is a part of the GU Ventures Incubator Program, all outside the submitted work. HZ has served at scientific advisory boards for Eisai, Denali, Roche Diagnostics, Wave, Samumed, Siemens Healthineers, Pinteon Therapeutics, Nervgen, AZTherapies and CogRx, has given lectures in symposia sponsored by Cellectricon, Fujirebio, Alzecure and Biogen, and is a co-founder of Brain Biomarker Solutions in Gothenburg AB (BBS), which is a part of the GU Ventures Incubator Program (outside submitted work). The other authors report a conflict of interest relevant to this research.

## CRediT authorship contribution statement

**Derek B. Archer:** Conceptualization, Methodology, Software, Validation, Formal analysis, Investigation, Writing - original draft, Writing - review & editing, Visualization. **Elizabeth E. Moore:** Validation, Writing - original draft, Writing - review & editing. **Ujwala Pamidimukkala:** Writing - original draft, Writing - review & editing. **Niranjana Shashikumar:** Methodology, Software, Validation, Data curation, Writing - review & editing. **Kimberly R. Pechman:** Data curation, Writing - review & editing. **Kaj Blennow:** Writing - review & editing. **Henrik Zetterberg:** Resources, Writing - review & editing. **Bennett A. Landman:** Writing - review & editing. **Timothy J. Hohman:** Resources, Writing - review & editing, Supervision, Funding acquisition. **Angela L. Jefferson:** Resources, Writing - review & editing, Supervision, Project administration, Funding acquisition. **Katherine A. Gifford:** Conceptualization, Methodology, Resources, Writing - original draft, Writing - review & editing, Supervision, Funding acquisition.
