# Supplementary material for: The relationship between white matter microstructure and self-perceived cognitive decline
Source: Neuroimage Clin. 2021 Aug 28;32:102794. doi: 10.1016/j.nicl.2021.102794 (PMC8414539; doi:10.1016/j.nicl.2021.102794)
Supplement: Supplementary data 1 [file mmc1.docx]

## Supplemental Table 1 – Participant Demographic and Clinical Characteristics in CSF Cohort

| **Measure** | | | **Cognitive Status** | | | **Test Statistic** | ***p*-value** |
| --- | --- | --- | --- | --- | --- | --- | --- |
|  |  |  | **All** | **Cognitively Unimpaired (CU)** | **MCI** |  |  |
| Demographics | | |  |  |  |  |  |
| Sample Size | | 104 | 64 | 30 | - | - |  |
| Age (yrs) | | 73 (6) | 72 (6) | 73 (6) | t=0.667 | 0.506 |  |
| Sex (% male) | | 74 | 77 | 70 | χ^2^=0.263 | 0.608 |  |
| Education (yrs) | | 16 (3) | 17 (3) | 15 (3) | t=2.726 | **0.007** |  |
| Race (% Non-Hispanic white) | | 93 | 94 | 93 | χ^2^=0.000 | 1.000 |  |
| Clinical Characteristics | | |  |  |  |  |  |
| MoCA | | | 26 (3) | 27 (2) | 23 (3) | t=6.723 | **<0.001** |
| *APOE* ε4 (% positive) | | 36 | 30 | 45 | χ^2^=1.894 | 0.169 |  |
| FSRP Score^a^ | | 12 (4) | 12 (4) | 12 (4) | t=0.843 | 0.400 |  |
| Systolic Blood Pressure (mmHg) | | 141 (16) | 139 (15) | 145 (16) | t=1.895 | 0.061 |  |
| Prevalent CVD (%) | | 4 | 6 | 0 | χ^2^=1.185 | 0.276 |  |
| GDS Score^b^ | | 2 (2) | 2 (2) | 3 (3) | t=2.824 | **0.006** |  |
| Hippocampal Volume (mm^3^) | | 7343 (797) | 7572 (736) | 6978 (763) | t=3.952 | **<0.001** |  |
| CSF Biomarkers^c^ | | |  |  |  |  |  |
| Aβ_42_ (pg/mL) | | 706 (241) | 760 (231) | 620 (234) | t=2.996 | **0.003** |  |
| T-tau (pg/mL) | | 422 (212) | 383 (180) | 486 (244) | H=4.416 | **0.036** |  |
| P-tau (pg/mL) | | 61 (26) | 57 (22) | 67 (29) | H=2.573 | 0.109 |  |
| Subjective Cognitive Decline Scores | | |  |  |  |  |  |
| Total Score | | 61 (22) | 52 (18) | 75 (21) | t=5.955 | **<0.001** |  |
| Executive Function | | 11 (5) | 9 (4) | 14 (5) | t=4.755 | **<0.001** |  |
| Language | | 14 (6) | 12 (4) | 17 (6) | H=20.547 | **<0.001** |  |
| Memory | | 36 (13) | 31 (11) | 45 (12) | t=5.849 | **<0.001** |  |
|  | | Values denoted as mean (standard deviation) or frequency. Abbreviations: *APOE* ε4, apolipoprotein E ε4; CVD, cardiovascular disease; FSRP, Framingham Stroke Risk Profile; GDS, Geriatric Depression Scale; MCI, mild cognitive impairment. p-values (p<0.05 bolded) were generated using a one-way analysis of variance for continuous variables and a chi-square test for categorical variables. ^a^A modified FSRP score was included in statistical models excluding points assigned to age. ^b^GDS score minus points from items 14, 26, 29, and 30. The adjusted range of this score is 0-26. ^c^A subset of 104 participants (64 CU, 30 MCI) had CSF biomarker data. Bolded values indicate p<0.05. | | | | | |

## Supplemental Table 2 -- White Matter Tract Microstructure Associations with Subjective Cognitive Decline Memory Subscore

|  | **Cingulum**  **Bundle** | **Fornix** | **ILF** | **IFG Pars Opercularis** | **IFG Pars Orbitalis** | **IFG Pars Triangularis** | **Tapetum** | **Medial Frontal Gyrus** | **Middle Frontal Gyrus** | **SLF** | **UF** |
| --- | --- | --- | --- | --- | --- | --- | --- | --- | --- | --- | --- |
|  | | | | | | | | | | | |
| **Free-water (FW)** | | | | | | | | | | | |
| **β** | 0.043 | 0.006 | 0.056 | 0.089 | 0.130 | 0.073 | 0.103 | 0.148 | 0.112 | 0.078 | 0.159 |
| **β_SE_** | 0.077 | 0.073 | 0.073 | 0.070 | 0.069 | 0.069 | 0.067 | 0.064 | 0.070 | 0.066 | 0.068 |
| **p-value** | 0.575 | 0.929 | 0.447 | 0.206 | 0.059 | 0.292 | 0.128 | **0.021** | 0.110 | 0.243 | **0.021** |
| **f^2^** | 0.001 | 0.000 | 0.003 | 0.007 | 0.016 | 0.005 | 0.010 | 0.024 | 0.011 | 0.006 | 0.024 |
|  | | | | | | | | | | | |
| **FW-corrected fractional anisotropy (FA_T_)** | | | | | | | | | | | |
| **β** | -0.158 | 0.034 | -0.195 | -0.139 | -0.130 | -0.146 | -0.097 | -0.145 | -0.152 | -0.117 | -0.133 |
| **β_SE_** | 0.060 | 0.064 | 0.059 | 0.055 | 0.059 | 0.056 | 0.058 | 0.061 | 0.057 | 0.057 | 0.059 |
| **p-value** | **0.009*** | 0.598 | **0.001*** | **0.012*** | **0.028*** | **0.010*** | 0.099 | **0.018*** | **0.008*** | **0.040** | **0.025*** |
| **f^2^** | 0.031 | 0.001 | 0.048 | 0.029 | 0.022 | 0.030 | 0.012 | 0.025 | 0.032 | 0.019 | 0.023 |
|  | | | | | | | | | | | |
| **FW-corrected mean diffusivity (MD_T_)** | | | | | | | | | | | |
| **β** | 0.182 | 0.055 | 0.138 | 0.110 | 0.018 | 0.124 | 0.087 | 0.138 | 0.105 | 0.067 | 0.019 |
| **β_SE_** | 0.056 | 0.068 | 0.054 | 0.058 | 0.056 | 0.058 | 0.056 | 0.054 | 0.058 | 0.056 | 0.059 |
| **p-value** | **0.001** | 0.420 | **0.012** | 0.057 | 0.751 | **0.033** | 0.121 | **0.011** | 0.069 | 0.235 | 0.745 |
| **f^2^** | 0.047 | 0.003 | 0.028 | 0.016 | 0.000 | 0.021 | 0.011 | 0.029 | 0.015 | 0.006 | 0.000 |
|  | | | | | | | | | | | |
| **FW-corrected axial diffusivity (AD_T_)** | | | | | | | | | | | |
| **β** | -0.047 | 0.026 | -0.104 | -0.073 | -0.123 | -0.101 | -0.014 | -0.092 | -0.103 | -0.077 | -0.112 |
| **β_SE_** | 0.067 | 0.067 | 0.062 | 0.056 | 0.056 | 0.057 | 0.056 | 0.059 | 0.057 | 0.060 | 0.063 |
| **p-value** | 0.482 | 0.703 | 0.097 | 0.196 | **0.028** | 0.078 | 0.797 | 0.120 | 0.073 | 0.205 | 0.075 |
| **f^2^** | 0.002 | 0.001 | 0.012 | 0.008 | 0.022 | 0.014 | 0.000 | 0.011 | 0.015 | 0.007 | 0.014 |
|  | | | | | | | | | | | |
| **FW-corrected radial diffusivity (RD_T_)** | | | | | | | | | | | |
| **β** | 0.189 | 0.050 | 0.191 | 0.144 | 0.127 | 0.162 | 0.100 | 0.170 | 0.155 | 0.098 | 0.096 |
| **β_SE_** | 0.054 | 0.062 | 0.054 | 0.055 | 0.054 | 0.054 | 0.055 | 0.055 | 0.054 | 0.054 | 0.055 |
| **p-value** | **0.001*** | 0.416 | **0.001*** | **0.010*** | **0.021*** | **0.003*** | 0.071 | **0.002*** | **0.005*** | 0.073 | 0.084 |
| **f^2^** | 0.055 | 0.003 | 0.055 | 0.030 | 0.024 | 0.040 | 0.015 | 0.043 | 0.036 | 0.014 | 0.013 |

Abbreviations: IFG, inferior frontal gyrus; ILF, inferior longitudinal fasciculus; SLF, superior longitudinal fasciculus; UF, uncinate fasciculus; β, beta-coefficient for variable of interest; β_SE_, standard error of the beta-coefficient. Bolded values indicate p<0.05, *p_FDR_<0.05.

## Supplemental Table 3 -- White Matter Tract Microstructure Associations with Subjective Cognitive Decline Executive Function Subscore

|  | **Cingulum**  **Bundle** | **Fornix** | **ILF** | **IFG Pars Opercularis** | **IFG Pars Orbitalis** | **IFG Pars Triangularis** | **Tapetum** | **Medial Frontal Gyrus** | **Middle Frontal Gyrus** | **SLF** | **UF** |
| --- | --- | --- | --- | --- | --- | --- | --- | --- | --- | --- | --- |
|  | | | | | | | | | | | |
| **Free-water (FW)** | | | | | | | | | | | |
| **β** | 0.070 | -0.006 | 0.037 | 0.033 | 0.071 | 0.025 | 0.085 | 0.082 | 0.036 | 0.018 | 0.101 |
| **β_SE_** | 0.076 | 0.072 | 0.073 | 0.070 | 0.069 | 0.069 | 0.067 | 0.064 | 0.070 | 0.066 | 0.068 |
| **p-value** | 0.362 | 0.938 | 0.606 | 0.635 | 0.300 | 0.716 | 0.208 | 0.203 | 0.608 | 0.790 | 0.139 |
| **f^2^** | 0.004 | 0.000 | 0.001 | 0.001 | 0.005 | 0.001 | 0.007 | 0.007 | 0.001 | 0.000 | 0.010 |
|  | | | | | | | | | | | |
| **FW-corrected fractional anisotropy (FA_T_)** | | | | | | | | | | | |
| **β** | -0.176 | -0.008 | -0.177 | -0.144 | -0.121 | -0.137 | -0.125 | -0.139 | -0.127 | -0.120 | -0.148 |
| **β_SE_** | 0.059 | 0.063 | 0.059 | 0.055 | 0.059 | 0.056 | 0.058 | 0.061 | 0.057 | 0.056 | 0.058 |
| **p-value** | **0.003*** | 0.896 | **0.003*** | **0.009*** | **0.040** | **0.015*** | **0.032*** | **0.023*** | **0.027*** | **0.034*** | **0.012*** |
| **f^2^** | 0.039 | 0.000 | 0.040 | 0.031 | 0.019 | 0.027 | 0.021 | 0.023 | 0.022 | 0.020 | 0.028 |
|  | | | | | | | | | | | |
| **FW-corrected mean diffusivity (MD_T_)** | | | | | | | | | | | |
| **β** | 0.099 | 0.080 | 0.111 | 0.115 | 0.056 | 0.125 | 0.063 | 0.154 | 0.121 | 0.047 | 0.054 |
| **β_SE_** | 0.057 | 0.068 | 0.054 | 0.057 | 0.055 | 0.058 | 0.056 | 0.053 | 0.057 | 0.056 | 0.058 |
| **p-value** | 0.082 | 0.242 | **0.043** | **0.046** | 0.312 | **0.032** | 0.265 | **0.004** | **0.035** | 0.404 | 0.354 |
| **f^2^** | 0.014 | 0.006 | 0.018 | 0.018 | 0.005 | 0.021 | 0.006 | 0.037 | 0.020 | 0.003 | 0.004 |
|  | | | | | | | | | | | |
| **FW-corrected axial diffusivity (AD_T_)** | | | | | | | | | | | |
| **β** | -0.124 | 0.016 | -0.112 | -0.070 | -0.101 | -0.091 | -0.065 | -0.089 | -0.080 | -0.092 | -0.115 |
| **β_SE_** | 0.066 | 0.067 | 0.062 | 0.056 | 0.056 | 0.057 | 0.056 | 0.058 | 0.057 | 0.060 | 0.063 |
| **p-value** | 0.061 | 0.813 | 0.073 | 0.211 | 0.070 | 0.113 | 0.246 | 0.130 | 0.162 | 0.125 | 0.067 |
| **f^2^** | 0.016 | 0.000 | 0.015 | 0.007 | 0.015 | 0.011 | 0.006 | 0.010 | 0.009 | 0.011 | 0.015 |
|  | | | | | | | | | | | |
| **FW-corrected radial diffusivity (RD_T_)** | | | | | | | | | | | |
| **β** | 0.157 | 0.087 | 0.169 | 0.149 | 0.137 | 0.157 | 0.135 | 0.179 | 0.153 | 0.091 | 0.126 |
| **β_SE_** | 0.054 | 0.061 | 0.054 | 0.055 | 0.054 | 0.054 | 0.054 | 0.054 | 0.054 | 0.054 | 0.055 |
| **p-value** | **0.004*** | 0.155 | **0.002*** | **0.007*** | **0.012*** | **0.004*** | **0.014*** | **0.001*** | **0.005*** | 0.093 | **0.023*** |
| **f^2^** | 0.038 | 0.009 | 0.043 | 0.033 | 0.029 | 0.038 | 0.027 | 0.048 | 0.036 | 0.013 | 0.024 |

Abbreviations: IFG, inferior frontal gyrus; ILF, inferior longitudinal fasciculus; SLF, superior longitudinal fasciculus; UF, uncinate fasciculus; β, beta-coefficient for variable of interest; β_SE_, standard error of the beta-coefficient. Bolded values indicate p<0.05, *p_FDR_<0.05.

## Supplemental Table 4 -- White Matter Tract Microstructure Associations with Subjective Cognitive Decline Language Subscore

|  | **Cingulum**  **Bundle** | **Fornix** | **ILF** | **IFG Pars Opercularis** | **IFG Pars Orbitalis** | **IFG Pars Triangularis** | **Tapetum** | **Medial Frontal Gyrus** | **Middle Frontal Gyrus** | **SLF** | **UF** |
| --- | --- | --- | --- | --- | --- | --- | --- | --- | --- | --- | --- |
|  | | | | | | | | | | | |
| **Free-water (FW)** | | | | | | | | | | | |
| **β** | 0.009 | 0.024 | 0.069 | 0.041 | 0.057 | 0.057 | 0.023 | 0.090 | 0.071 | 0.069 | 0.099 |
| **β_SE_** | 0.077 | 0.073 | 0.073 | 0.071 | 0.070 | 0.069 | 0.068 | 0.065 | 0.071 | 0.067 | 0.069 |
| **p-value** | 0.906 | 0.740 | 0.351 | 0.563 | 0.412 | 0.416 | 0.740 | 0.167 | 0.315 | 0.307 | 0.154 |
| **f^2^** | 0.000 | 0.000 | 0.004 | 0.001 | 0.003 | 0.003 | 0.000 | 0.009 | 0.005 | 0.005 | 0.009 |
|  | | | | | | | | | | | |
| **FW-corrected fractional anisotropy (FA_T_)** | | | | | | | | | | | |
| **β** | -0.143 | -0.064 | -0.132 | -0.126 | -0.128 | -0.144 | -0.117 | -0.143 | -0.128 | -0.103 | -0.110 |
| **β_SE_** | 0.061 | 0.064 | 0.061 | 0.056 | 0.059 | 0.057 | 0.059 | 0.062 | 0.058 | 0.057 | 0.060 |
| **p-value** | **0.020*** | 0.322 | **0.031*** | **0.025*** | **0.033*** | **0.012*** | **0.048** | **0.021*** | **0.028*** | 0.073 | 0.066 |
| **f^2^** | 0.025 | 0.004 | 0.021 | 0.023 | 0.021 | 0.029 | 0.018 | 0.024 | 0.022 | 0.014 | 0.015 |
|  | | | | | | | | | | | |
| **FW-corrected mean diffusivity (MD_T_)** | | | | | | | | | | | |
| **β** | 0.106 | 0.065 | 0.039 | 0.086 | 0.044 | 0.070 | 0.068 | 0.108 | 0.068 | -0.041 | 0.040 |
| **β_SE_** | 0.058 | 0.069 | 0.056 | 0.058 | 0.056 | 0.059 | 0.057 | 0.055 | 0.058 | 0.057 | 0.059 |
| **p-value** | 0.067 | 0.350 | 0.489 | 0.141 | 0.430 | 0.236 | 0.230 | **0.049** | 0.247 | 0.469 | 0.500 |
| **f^2^** | 0.015 | 0.004 | 0.002 | 0.010 | 0.003 | 0.006 | 0.006 | 0.017 | 0.006 | 0.002 | 0.002 |
|  | | | | | | | | | | | |
| **FW-corrected axial diffusivity (AD_T_)** | | | | | | | | | | | |
| **β** | -0.090 | -0.030 | -0.110 | -0.065 | -0.095 | -0.104 | -0.053 | -0.089 | -0.088 | -0.133 | -0.093 |
| **β_SE_** | 0.067 | 0.068 | 0.063 | 0.057 | 0.056 | 0.058 | 0.057 | 0.059 | 0.058 | 0.060 | 0.064 |
| **p-value** | 0.180 | 0.655 | 0.083 | 0.251 | 0.093 | 0.073 | 0.345 | 0.136 | 0.127 | **0.028** | 0.146 |
| **f^2^** | 0.008 | 0.001 | 0.014 | 0.006 | 0.013 | 0.014 | 0.004 | 0.010 | 0.010 | 0.022 | 0.009 |
|  | | | | | | | | | | | |
| **FW-corrected radial diffusivity (RD_T_)** | | | | | | | | | | | |
| **β** | 0.141 | 0.099 | 0.095 | 0.119 | 0.121 | 0.123 | 0.124 | 0.141 | 0.114 | 0.040 | 0.099 |
| **β_SE_** | 0.055 | 0.062 | 0.056 | 0.056 | 0.055 | 0.055 | 0.055 | 0.056 | 0.055 | 0.055 | 0.056 |
| **p-value** | **0.011*** | 0.113 | 0.091 | **0.035** | **0.028*** | **0.026*** | **0.025*** | **0.012*** | **0.041** | 0.464 | 0.079 |
| **f^2^** | 0.030 | 0.011 | 0.013 | 0.020 | 0.022 | 0.022 | 0.023 | 0.029 | 0.019 | 0.002 | 0.014 |

Abbreviations: IFG, inferior frontal gyrus; ILF, inferior longitudinal fasciculus; SLF, superior longitudinal fasciculus; UF, uncinate fasciculus; β, beta-coefficient for variable of interest; β_SE_, standard error of the beta-coefficient. Bolded values indicate p<0.05, *p_FDR_<0.05.
